# *N*-Benzyl-2-propynamide

**DOI:** 10.1107/S160053680901455X

**Published:** 2009-04-25

**Authors:** Mei-Mei Chen, Yu-Xing Gao, Hai-Yan Wang, Da-Xiong Han, Yu-Fen Zhao

**Affiliations:** a Department of Pharmacy, Medical College of Xiamen University, Xiamen 361005, People’s Republic of China; b Department of Chemistry and the Key Laboratory for Chemical Biology of Fujian Province, College of Chemistry and Chemical Engineering, Xiamen University, Xiamen 361005, People’s Republic of China; c The Third Institute of Oceanography, State Oceanic Administration of China, Xiamen 361005, People’s Republic of China

## Abstract

Pale-yellow crystals of the title compound, C_10_H_9_NO, have been obtained by the reaction of benzyl­amine and methyl propiolate. Weak inter­molecular hydrogen bonding is observed between acetyl­enic H and carbonyl O atoms. The crystal packing is stabilized by these C—H⋯O and by N—H⋯O inter­molecular hydrogen-bonding inter­actions.

## Related literature

The title compound was synthesized using a similar synthetic method to that described by Williamson *et al.* (1994 ▶). For the synthesis of triazole derivatives, see: Katritzky & Singh (2002 ▶). For the structure of the methyl analogue of the title compound, see: Leiserowitz & Tuval (1978 ▶). For the program *ROTAX*, used to investigate possible pseudo-merohedral twinning, see: Parsons & Gould (2003 ▶).
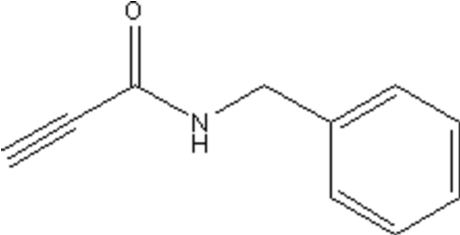


## Experimental

### 

#### Crystal data

C_10_H_9_NO
*M* *_r_* = 159.18
Monoclinic, 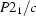

*a* = 9.495 (2) Å
*b* = 10.703 (2) Å
*c* = 8.9120 (19) Å
β = 101.637 (3)°
*V* = 887.1 (3) Å^3^
*Z* = 4
Mo *K*α radiation
μ = 0.08 mm^−1^
*T* = 173 K
0.57 × 0.30 × 0.30 mm

#### Data collection

Bruker SMART APEX area-detector diffractometer
Absorption correction: multi-scan (*SADABS*; Bruker, 2001 ▶) *T*_min_ = 0.848, *T*_max_ = 1.000 (expected range = 0.828–0.977)
5825 measured reflections
1550 independent reflections
1510 reflections with *I* > 2σ(*I*)
*R*_int_ = 0.030

#### Refinement

*R*[*F* ^2^ > 2σ(*F* ^2^)] = 0.070
*wR*(*F* ^2^) = 0.221
*S* = 1.26
1550 reflections
113 parameters
H atoms treated by a mixture of independent and constrained refinement
Δρ_max_ = 0.45 e Å^−3^
Δρ_min_ = −0.23 e Å^−3^

### 

Data collection: *SMART* (Bruker, 2001 ▶); cell refinement: *SAINT* (Bruker, 2001 ▶); data reduction: *SAINT*; program(s) used to solve structure: *SHELXS97* (Sheldrick, 2008 ▶); program(s) used to refine structure: *SHELXL97* (Sheldrick, 2008 ▶); molecular graphics: *ORTEP-3 for Windows* (Farrugia, 1997 ▶); software used to prepare material for publication: *SHELXL97*.

## Supplementary Material

Crystal structure: contains datablocks I, global. DOI: 10.1107/S160053680901455X/zl2187sup1.cif

Structure factors: contains datablocks I. DOI: 10.1107/S160053680901455X/zl2187Isup2.hkl

Additional supplementary materials:  crystallographic information; 3D view; checkCIF report

## Figures and Tables

**Table 1:** Hydrogen-bond geometry (Å, °)

*D*—H⋯*A*	*D*—H	H⋯*A*	*D*⋯*A*	*D*—H⋯*A*
N1—H1*A*⋯O1^i^	0.88	1.99	2.839 (3)	163
C1—H1⋯O1^ii^	0.93 (4)	2.17 (4)	3.105 (4)	176 (3)

Symmetry codes: (i) 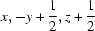; (ii) 
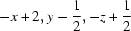
.

**Fig. 1.**
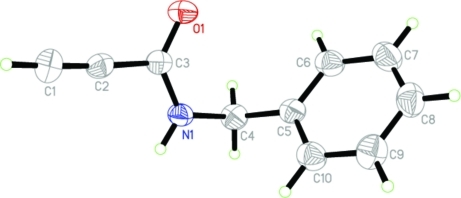
The molecular structure of the compound with 50% probability displacement ellipsoids (arbitrary spheres for H atoms).

**Fig. 2.**
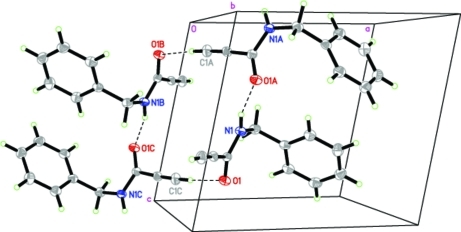
Part of the packing of the title compound. Intermolecular hydrogen bonds are represented by dashed lines.

## supplementary crystallographic information

### Comment

The title compound is a terminal alkyne, which is an intermediate in the synthesis of triazole derivatives (Katritzky *et al.*, 2002).

The molecular structure of the title compound is shown in Fig. 1. The bond lengths and bond angles in the compound are comparable to those in the structure of the methyl analogue (Leiserowitz *et al.*, 1978). The atoms C1, C2, C3, O1, N1 and C4 of the title compound are nearly in a plane, and the r.m.s. deviation of these atoms from their mean plane is 0.007 Å. The dihedral angle between the plane of C5 and the phenyl ring and the mean plane of C1 to C4 and N1 is 76.8 (2)°. Hydrogen bonding plays a significant role in stabilizing the crystal structure; see Table 1 for geometric parameters and symmetry operations. The most prominent link occurs between the acylamide O and the N atoms, to form chains along the *b* axis. Weak intermolecular hydrogen bonding is observed between the alkyne H and the carbonyl O atoms (table 1). Molecules are connected into a double chain by C—H···O and N—H···O intermolecular hydrogen-bonding interactions (Figure 2).

### Experimental

The title compound was synthesized using a similar synthetic method as for the preparation of 1-(pyrrolidin-1-yl)prop-2-yn-1-one (Williamson *et al.*, 1994). To a solution of benzyl amine (1.07 g, 10 mmol) in methanol (4 ml) was slowly added methyl propiolate (0.84 g, 10 mmol) at 195 K with stirring. After addition of the propiolate, the stirring was continued for 10 h and then the mixture warmed to 248 K for 5 h. The reaction was quenched with a saturated NH_4_Cl solution (12 ml) and extracted with ethyl acetate. The organic layer was washed with brine, dried over anhydrous MgSO_4_, concentrated under vacuum and the crude product was purified by column chromatography (petroleum ether: ethyl acetate, 2:1) to give the title compound as a pale yellow solid in 72% yield. Single crystals of the title compound were grown in a petroleum ether/ethyl acetate solution (*v*/*v* = 5:1) by slow evaporation.

### Refinement

All non-hydrogen atoms were refined anisotropically. The acetylenic H atom was located from a difference Fourier map and both the position and isotropic thermal parameter were freely refined. The remaining H atoms were placed in ideal positions and refined *via* a riding model with N-H distances of 0.88, C-H_methyelene_ = 0.99 and C-H_aromatic_ = 0.95 Å and U_iso_ = 1.2 U_eq_(C,N). Torsion angles were refined to fit the electron density. The metric parameters suggest the possibility of pseudo-merohedral twinning by a two fold rotation around either the *a* or the *c* axis. Application of the respective twin law of (-1 0 - 0.43, 0 1 0, 0 0 1), obtained using the program Rotax (Parsons & Gould, 2003)) however indicated that the crystal at hand was not twinned.

### Crystal data

**Table:**

C_10_H_9_NO	*F*(000) = 336
*M**_r_* = 159.18	*D*_x_ = 1.192 Mg m^−^^3^
Monoclinic, *P*2_1_/*c*	Mo *K*α radiation, λ = 0.71073 Å
Hall symbol: -P 2ybc	Cell parameters from 4373 reflections
*a* = 9.495 (2) Å	θ = 2.2–28.3°
*b* = 10.703 (2) Å	µ = 0.08 mm^−^^1^
*c* = 8.9120 (19) Å	*T* = 173 K
β = 101.637 (3)°	Chunk, pale yellow
*V* = 887.1 (3) Å^3^	0.57 × 0.30 × 0.30 mm
*Z* = 4	

### Data collection

**Table:**

Bruker APEX area-detector diffractometer	1550 independent reflections
Radiation source: fine-focus sealed tube	1510 reflections with *I* > 2σ(*I*)
graphite	*R*_int_ = 0.030
φ and ω scans	θ_max_ = 25.0°, θ_min_ = 2.9°
Absorption correction: multi-scan (*SADABS*; Bruker, 2001)	*h* = −11→11
*T*_min_ = 0.848, *T*_max_ = 1.000	*k* = −12→12
5825 measured reflections	*l* = −10→10

### Refinement

**Table:**

Refinement on *F*^2^	Primary atom site location: structure-invariant direct methods
Least-squares matrix: full	Secondary atom site location: difference Fourier map
*R*[*F*^2^ > 2σ(*F*^2^)] = 0.070	Hydrogen site location: inferred from neighbouring sites
*wR*(*F*^2^) = 0.221	H atoms treated by a mixture of independent and constrained refinement
*S* = 1.26	*w* = 1/[σ^2^(*F*_o_^2^) + (0.0874*P*)^2^ + 1.0844*P*] where *P* = (*F*_o_^2^ + 2*F*_c_^2^)/3
1550 reflections	(Δ/σ)_max_ < 0.001
113 parameters	Δρ_max_ = 0.45 e Å^−^^3^
0 restraints	Δρ_min_ = −0.23 e Å^−^^3^

### Special details

**Table:**

Geometry. All e.s.d.'s (except the e.s.d. in the dihedral angle between two l.s. planes) are estimated using the full covariance matrix. The cell e.s.d.'s are taken into account individually in the estimation of e.s.d.'s in distances, angles and torsion angles; correlations between e.s.d.'s in cell parameters are only used when they are defined by crystal symmetry. An approximate (isotropic) treatment of cell e.s.d.'s is used for estimating e.s.d.'s involving l.s. planes.
Refinement. Refinement of *F*^2^ against ALL reflections. The weighted *R*-factor *wR* and goodness of fit *S* are based on *F*^2^, conventional *R*-factors *R* are based on *F*, with *F* set to zero for negative *F*^2^. The threshold expression of *F*^2^ > σ(*F*^2^) is used only for calculating *R*-factors(gt) *etc*. and is not relevant to the choice of reflections for refinement. *R*-factors based on *F*^2^ are statistically about twice as large as those based on *F*, and *R*- factors based on ALL data will be even larger.

### Fractional atomic coordinates and isotropic or equivalent isotropic displacement parameters (Å^2^)

**Table:**

	*x*	*y*	*z*	*U*_iso_*/*U*_eq_	
O1	0.8163 (2)	0.3454 (2)	0.1877 (2)	0.0356 (6)	
N1	0.7947 (3)	0.3179 (2)	0.4330 (3)	0.0323 (7)	
H1A	0.8203	0.2728	0.5166	0.039*	
C1	1.0048 (4)	0.0859 (3)	0.3289 (4)	0.0401 (8)	
C2	0.9317 (3)	0.1756 (3)	0.3242 (3)	0.0317 (7)	
C3	0.8422 (3)	0.2867 (3)	0.3095 (3)	0.0290 (7)	
C4	0.7008 (4)	0.4254 (3)	0.4350 (4)	0.0365 (8)	
H4A	0.7389	0.4971	0.3855	0.044*	
H4B	0.7015	0.4487	0.5427	0.044*	
C5	0.5485 (3)	0.4009 (3)	0.3545 (3)	0.0323 (7)	
C6	0.4870 (4)	0.4688 (3)	0.2258 (4)	0.0413 (8)	
H6A	0.5418	0.5309	0.1873	0.050*	
C7	0.3471 (4)	0.4469 (4)	0.1532 (4)	0.0483 (9)	
H7A	0.3058	0.4943	0.0653	0.058*	
C8	0.2665 (4)	0.3569 (4)	0.2069 (4)	0.0466 (9)	
H8A	0.1702	0.3415	0.1559	0.056*	
C9	0.3268 (4)	0.2895 (3)	0.3353 (4)	0.0464 (9)	
H9A	0.2715	0.2277	0.3736	0.056*	
C10	0.4673 (4)	0.3113 (3)	0.4089 (4)	0.0411 (8)	
H10A	0.5081	0.2643	0.4974	0.049*	
H1	1.061 (4)	0.014 (4)	0.328 (4)	0.050 (11)*	

### Atomic displacement parameters (Å^2^)

**Table:**

	*U*^11^	*U*^22^	*U*^33^	*U*^12^	*U*^13^	*U*^23^
O1	0.0461 (13)	0.0335 (12)	0.0286 (12)	−0.0005 (10)	0.0112 (9)	0.0028 (9)
N1	0.0378 (14)	0.0352 (14)	0.0246 (13)	0.0058 (11)	0.0076 (10)	0.0010 (10)
C1	0.0344 (17)	0.0376 (19)	0.049 (2)	0.0016 (16)	0.0091 (14)	−0.0036 (15)
C2	0.0313 (16)	0.0355 (17)	0.0297 (16)	−0.0050 (13)	0.0091 (12)	−0.0018 (12)
C3	0.0285 (15)	0.0301 (15)	0.0275 (15)	−0.0076 (12)	0.0038 (11)	−0.0021 (12)
C4	0.0432 (18)	0.0323 (16)	0.0342 (17)	0.0031 (14)	0.0086 (13)	−0.0064 (13)
C5	0.0406 (17)	0.0284 (15)	0.0292 (15)	0.0061 (13)	0.0104 (12)	−0.0050 (12)
C6	0.050 (2)	0.0380 (18)	0.0368 (18)	0.0070 (15)	0.0116 (15)	0.0024 (14)
C7	0.051 (2)	0.054 (2)	0.0379 (19)	0.0174 (18)	0.0061 (16)	0.0028 (16)
C8	0.0387 (18)	0.053 (2)	0.046 (2)	0.0078 (16)	0.0040 (15)	−0.0089 (16)
C9	0.043 (2)	0.0391 (19)	0.057 (2)	−0.0032 (15)	0.0112 (16)	−0.0014 (16)
C10	0.0459 (19)	0.0358 (17)	0.0412 (18)	0.0040 (14)	0.0080 (15)	0.0049 (14)

### Geometric parameters (Å, °)

**Table:**

O1—C3	1.235 (4)	C5—C6	1.384 (5)
N1—C3	1.314 (4)	C6—C7	1.376 (5)
N1—C4	1.458 (4)	C6—H6A	0.9500
N1—H1A	0.8800	C7—C8	1.374 (6)
C1—C2	1.180 (5)	C7—H7A	0.9500
C1—H1	0.93 (4)	C8—C9	1.376 (5)
C2—C3	1.453 (4)	C8—H8A	0.9500
C4—C5	1.502 (5)	C9—C10	1.383 (5)
C4—H4A	0.9900	C9—H9A	0.9500
C4—H4B	0.9900	C10—H10A	0.9500
C5—C10	1.378 (5)		
			
C3—N1—C4	121.7 (3)	C6—C5—C4	120.6 (3)
C3—N1—H1A	119.2	C7—C6—C5	120.4 (3)
C4—N1—H1A	119.2	C7—C6—H6A	119.8
C2—C1—H1	178 (2)	C5—C6—H6A	119.8
C1—C2—C3	176.9 (3)	C8—C7—C6	120.6 (3)
O1—C3—N1	124.5 (3)	C8—C7—H7A	119.7
O1—C3—C2	120.3 (3)	C6—C7—H7A	119.7
N1—C3—C2	115.2 (3)	C7—C8—C9	119.3 (3)
N1—C4—C5	112.8 (2)	C7—C8—H8A	120.4
N1—C4—H4A	109.0	C9—C8—H8A	120.4
C5—C4—H4A	109.0	C8—C9—C10	120.4 (3)
N1—C4—H4B	109.0	C8—C9—H9A	119.8
C5—C4—H4B	109.0	C10—C9—H9A	119.8
H4A—C4—H4B	107.8	C5—C10—C9	120.4 (3)
C10—C5—C6	119.0 (3)	C5—C10—H10A	119.8
C10—C5—C4	120.4 (3)	C9—C10—H10A	119.8
			
C4—N1—C3—O1	2.1 (5)	C5—C6—C7—C8	−0.3 (5)
C4—N1—C3—C2	−178.4 (3)	C6—C7—C8—C9	0.6 (5)
C3—N1—C4—C5	76.0 (4)	C7—C8—C9—C10	−0.5 (5)
N1—C4—C5—C10	63.6 (4)	C6—C5—C10—C9	0.3 (5)
N1—C4—C5—C6	−117.3 (3)	C4—C5—C10—C9	179.4 (3)
C10—C5—C6—C7	−0.2 (5)	C8—C9—C10—C5	0.0 (5)
C4—C5—C6—C7	−179.3 (3)		

### Hydrogen-bond geometry (Å, °)

**Table:**

*D*—H···*A*	*D*—H	H···*A*	*D*···*A*	*D*—H···*A*
N1—H1A···O1^i^	0.88	1.99	2.839 (3)	163
C1—H1···O1^ii^	0.93 (4)	2.17 (4)	3.105 (4)	176 (3)

Symmetry codes: (i) *x*, −*y*+1/2, *z*+1/2; (ii) −*x*+2, *y*−1/2, −*z*+1/2.
